# Noninvasive in vivo imaging of NF-κB activation predicts immunotherapy response in solid tumors

**DOI:** 10.1038/s44303-026-00173-8

**Published:** 2026-05-20

**Authors:** Dimitri Stowbur, Roman Mehling, Philipp Knopf, Barbara Francisca Schörg, Dominik Sonanini, Danielle Arnold-Schild, Irene Gonzalez-Menendez, Leticia Quintanilla-Martinez, Birgit Fehrenbacher, Martin Schaller, Harald Carlsen, Snehlata Kumari, Bernd Jürgen Pichler, Manfred Kneilling

**Affiliations:** 1 Werner Siemens Imaging Center, Department of Preclinical Imaging and Radiopharmacy, Eberhard Karls University Tübingen, Tübingen, Germany; 2 Cluster of Excellence iFIT (EXC 2180) “Image Guided and Functionally Instructed Tumor Therapies”, Tübingen, Germany; 3 Internal Medicine VIII, University Hospital of Tübingen, Tübingen, Germany; 4 Institute of Immunology, University Medical Center Mainz, Mainz, Germany; 5 Core Facility Histology, Faculty of Medicine Tübingen, University Hospital Tübingen, Tübingen, Germany; 6 Department of Dermatology, Eberhard Karls University, Tübingen, Germany; 7 Department of Chemistry, Biotechnology and Food Science, Norwegian University of Life Sciences, Ås, Norway; 8 Frazer Institute, Faculty of Health, Medicine and Behavioural Science, The University of Queensland, Brisbane, QLD Australia; 9 Dermatology Research Centre, The University of Queensland, Brisbane, QLD Australia; 10 German Cancer Consortium (DKTK) and German Cancer Research Center, Heidelberg, Germany

**Keywords:** Cancer, Immunology, Oncology

## Abstract

Cancer immunotherapy improves survival, yet many patients exhibit primary or acquired resistance. We established a combinatorial immunotherapy (COMBO) consisting of tumor antigen-specific Th1 cells and dual immune checkpoint blockade and aimed to noninvasively identify sites of immune activation associated with therapeutic response. Using ^NF-κB^Luc-reporter mice, we longitudinally monitored NF-κB activation by using in vivo bioluminescence imaging in the tumor microenvironment (TME) and bone marrow (BM) of mice bearing OVA-expressing MC38 adenocarcinoma (responder) or B16 melanoma (non-responder). COMBO treatment induced tumor regression in OVA-MC38 but not OVA-B16 tumors. Responsive tumors showed significantly increased NF-κB activation in the TME, whereas resistant melanomas displayed no therapy-induced NF-κB activation. In contrast, BM NF-κB activity was reduced upon COMBO treatment in both models. Immunofluorescence and flow cytometry analyses revealed NF-κB activation in tumor-infiltrating MPO⁺ neutrophils and a concomitant reduction of CD11b⁺Gr-1^high^ neutrophils in the BM in OVA-MC38 bearing mice, suggesting therapy-driven myeloid cell egress. Thus, therapeutic efficacy strongly correlated with NF-κB activation within the TME, while systemic BM changes reflected immune mobilization. Longitudinal imaging of NF-κB activity may enable early discrimination between therapy-sensitive and -resistant tumors in preclinical models.

## Introduction

Cancer immunotherapy strategies have become a cornerstone of routine clinical practice.^1^ Among these, immune checkpoint inhibitors (ICIs) have emerged as a powerful tool, utilizing immune checkpoint-specific antibodies to target key molecules such as cytotoxic T-lymphocyte-associated protein-4 (CTLA-4; ipilimumab) or programmed cell death protein-1 (PD-1; nivolumab and pembrolizumab). Therapeutic blockade of the PD-1 (programmed death-ligand 1)/PD-L1 axis by ICIs is currently approved as a first-line treatment for patients with metastatic melanoma, non-small cell lung cancer, head and neck cancer, Merkel cell carcinoma, colorectal cancer, etc.^2–4^ Another promising immune checkpoint target is lymphocyte-activation gene 3 (LAG-3), which is expressed on the surface of activated T cells. By binding to MHC class II molecules with an affinity up to four times greater than that of the T-cell receptor (TCR), LAG-3 effectively outcompetes TCR engagement and thereby exerts a potent inhibitory effect on T-cell activation and function.^5^ Despite the remarkable progress in immune checkpoint inhibitor (ICI)-based cancer immunotherapy, substantial variability in treatment efficacy persists, largely because of tumor entity-specific factors and heterogeneous PD-L1 expression. Moreover, the differences in treatment efficacy between individual patients with identical tumor entities and PD-L1 expression patterns remain poorly understood.^6^ In patients with advanced non-small cell lung cancer (NSCLC) with PD-L1 expression in at least 50% of tumor cells, compared with platinum-based chemotherapy, pembrolizumab treatment resulted in significantly prolonged progression-free survival and overall survival and was associated with a lower incidence of treatment-related adverse events.^7^ Furthermore, compared with ipilimumab, pembrolizumab significantly improved long-term survival in advanced melanoma patients, with a median progression-free survival of 9.4 months versus 3.8 months and 10-year overall survival rates of 34.0% versus 23.6%.^8^

Hence, understanding the molecular mechanisms underlying primary and secondary resistance to cancer immunotherapy is urgently needed in cancer research.^9,10^ It is essential to identify patients who are likely to benefit from cancer immunotherapy and stratify therapy-resistant patients to provide an additional standard of care.^11^ These early interventions minimize severe adverse side effects for nonresponders and optimize positive patient outcomes for responders. Hence, the financial and health burden on both patients and healthcare systems can be reduced.

Nuclear factor kappa-light-chain-enhancer of activated B cells (NF-κB) is a transcription factor that plays a critical role in regulating immune responses, inflammation, cell survival, and other cellular processes important for both tumor growth and tumor eradication.^12^ NF-κB consists of five structurally related members, namely, NF-κB 1 (p50), NF-κB 2 (p52), RelA (p65), RelB and c-Rel. As hetero- or homodimers, these factors bind to a specific DNA sequence—the κB site—and thereby trigger the transcription of NF-κB-specific genes.^13^ Carlsen et al. generated a transgenic ^NF-κB^Luc reporter mouse that expresses firefly luciferase under the control of three NF-κB response elements.^13^ Upon activation, NF-κB transcription factors bind to their specific DNA response elements (κB sites) and thereby induce transcription of the luciferase reporter gene. Upon the administration of luciferin, luciferase gene activity can be measured using noninvasive in vivo imaging of NF-κB activation by bioluminescence optical imaging (BLI).^14^ We have recently studied NF-κB activation in ^NF-κB^Luc-reporter mice with acute and chronic T-cell-mediated cutaneous delayed-type hypersensitivity reactions upon anti-inflammatory therapy.^15^ Thus, ^NF-κB^Luc-reporter mice represent an established and reliable innovative tool for monitoring NF-κB activation upon cancer immunotherapy in a holistic manner. This is particularly important, as how cancer immunotherapy modulates NF-κB activation in the TME and in the lymphatic organs of tumor-bearing experimental mice is not fully understood.

On the one hand, NF-κB activation may act as a good prognostic factor by driving immune activation in dendritic cells, macrophages, and T cells; enhancing antigen presentation; and promoting the release of proinflammatory cytokines (e.g., TNF and IL-12) and improving therapeutic responses to ICI therapy by increasing effector immune cell infiltration.^16,17^

On the other hand, NF-κB activation can serve as a poor prognostic factor, as its chronic activation in tumors and stromal cells promotes angiogenesis and immune suppression through M2 macrophages, MDSCs, and Tregs and enhances tumor survival and therapy resistance.^18–20^

Preclinically, various ICI-sensitive or ICI-responsive experimental tumor models have been established to study specific differences and mechanisms to overcome therapy resistance.^21–23^ In recent preclinical studies, we intensively investigated responsive and resistant syngeneic tumor models, including MC38 and CT26 colon adenocarcinoma as well as B16F10 melanoma and 4T1 mammary carcinoma cells, and thereby identified a novel acidosis-related tumor immune escape mechanism.^21^ In ovalbumin (OVA)-expressing MC38 or B16F10 tumor-bearing mice, ICI therapy can be enhanced by adoptive transfer of OVA-specific CD4^+^ T cells derived from OVA-specific T-cell receptor transgenic OT-II mice.^24^ Combined immunotherapies, such as OVA-specific CD4^+^ T-cell transfer and anti-PD-L1 ICI treatment, have been shown to reduce the metastatic burden in an OVA-B16 tumor mouse model.^25^ However, to our knowledge, it has never been evaluated in OVA-MC38 tumors. In a head-to-head comparison of OVA-expressing tumor models, Escobar et al. demonstrated that adoptively transferred OT-I CD8⁺ T cells combined with anti-PD-L1 and anti-T-cell immunoglobulin and mucin domain-containing protein 3 (TIM-3) monoclonal antibodies (mAbs) promoted T-cell expansion and tumor regression in highly immunogenic **OVA-MC38** tumors. In contrast, the same regimen achieved only modest control in poorly immunogenic **OVA-B16** tumors.^26^

In this study, we investigated tumor-bearing experimental mice with either sensitive OVA-MC38 colon adenocarcinoma or resistant OVA-B16 melanomas. The experimental mice underwent COMBO treatment, previously evaluated in RIP-Tag2 mice with progressed insular cell carcinomas, consisting of low-dose whole-body irradiation (2 Gy), adoptive transfer of OVA-T_H_1 cells, and immune checkpoint-inhibiting mAbs targeting PD-L1 and LAG-3.^27^ As it remains unclear how NF-κB activation differs between cancer immunotherapy-sensitive and immunotherapy-resistant tumor models, we aimed to determine whether holistic, noninvasive in vivo monitoring of NF-κB activation in the TME (stroma cells and resident or infiltrating endogenous immune cells) and in the BM can predict tumor responsiveness to our combined cancer immunotherapy regimen.

## Results

### COMBO treatment is highly effective against solid OVA-MC38 tumors

**Fig. 1:**
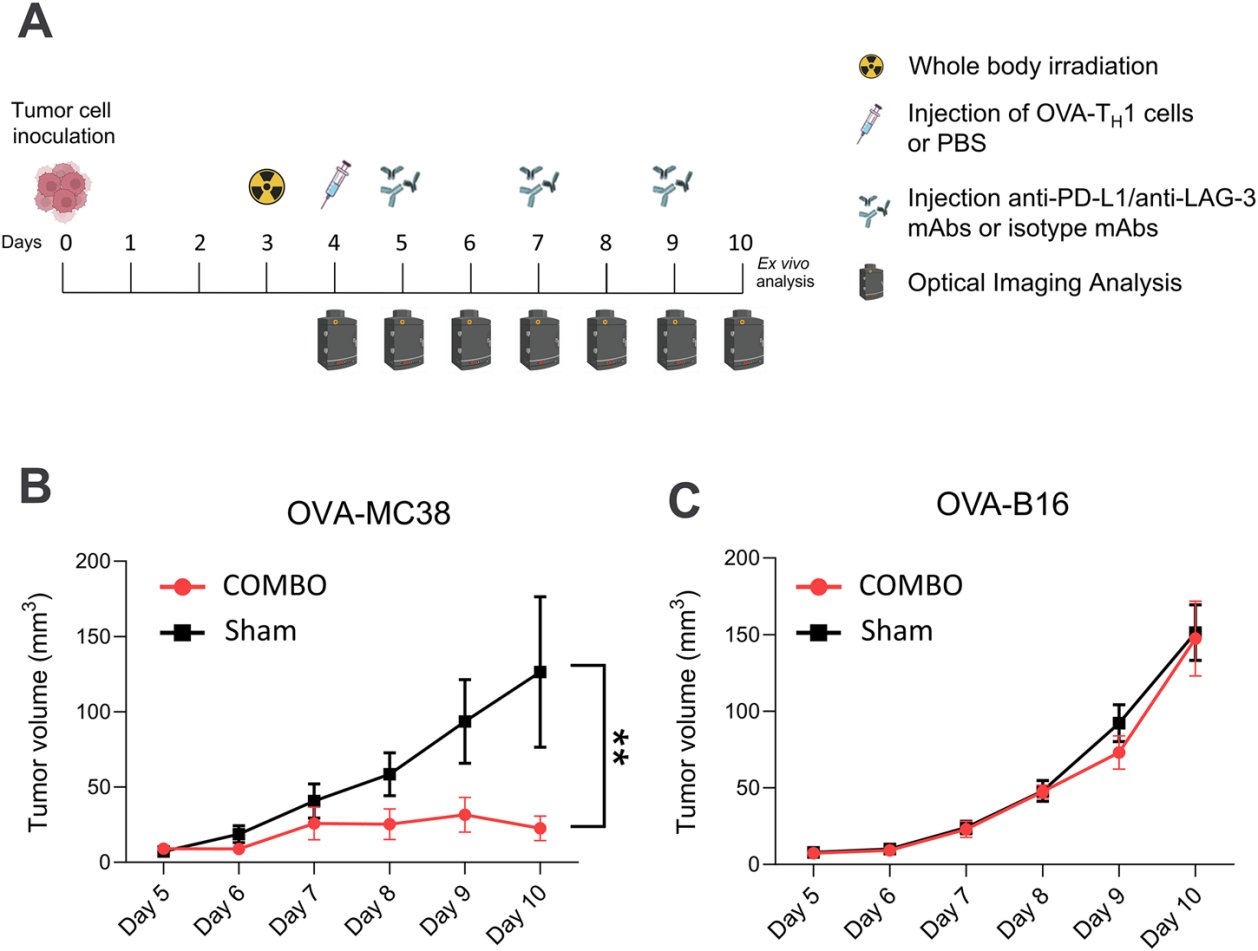
COMBO treatment suppresses tumor growth in therapy-responsive OVA-MC38 tumors. **A** Schematic illustration of the experimental design. On day 0, ^NF-κB^Luc reporter mice were shaved and subsequently inoculated with either OVA-B16 or OVA-MC38 tumor cells. Three days after tumor inoculation (day 3), all the mice underwent low-dose whole-body irradiation. After 24 h (day 4), the experimental mice were separated into two cohorts: The COMBO treatment group received an *i.p*. administration of OVA-T_H_1 cells, and the sham treatment group was injected with PBS (*i.p*.). Twenty-four hours later (day 5) and at days 7 and 9, the COMBO treatment group was injected *i.p*. with anti-PD-L1 and anti-LAG-3 mAbs, and the sham-treatment group was injected with the isotype mAbs. All the mice were sacrificed on day 10 for ex vivo analysis. Created with BioRender.com **B** Tumor volume (mean ± SEM) of OVA-MC38 (*n* = 4–5; sham treatment: *n* = 4; COMBO treatment: *n* = 5) and (**C**) OVA-B16 (*n* = 7 per treatment group). Tumor volumes were determined daily using a caliper until the end of the experiment. The difference in tumor volume on each day between the COMBO-treated group and the sham-treated group was compared using 2-way ANOVA and Sidak’s multiple comparison test. The data are presented as the mean ± SEM. The relative tumor volumes are shown in Supplementary Fig. 1. *n* = 4–6.

In this study, we monitored tumor growth upon combined cancer immunotherapy in OVA-MC38 or OVA-B16 tumor-bearing ^NF-κB^Luc-reporter mice (Fig. 1A**)**. Four days after tumor cell inoculation, the mice underwent COMBO treatment composed of 2 Gy of whole-body radiation, OVA-Th1 cells, and αPD-L1- and αLAG-3-specific mAbs. Sham-treated tumor-bearing mice received 2 Gy of whole-body radiation, PBS or isotype mAbs instead of OVA-Th1 cells or ICI. By day 7 postinoculation (day 4 of treatment), compared with sham-treated control mice, COMBO-treated OVA-MC38 tumor-bearing mice showed marked tumor growth inhibition. At the endpoint (day 10), COMBO therapy significantly reduced tumor volume (23 ± 8 mm³, *n* = 4) versus that observed in sham-treated mice (126 ± 50 mm³, *n* = 5; *p* = 0.0053; Fig. 1B). In contrast, COMBO treatment showed no therapeutic effect in OVA-B16 tumor-bearing experimental mice compared with that observed in sham-treated littermates (Fig. 1C), confirming the efficacy of COMBO treatment for therapy-sensitive tumors.

### COMBO treatment induces NF-κB activation in the tumor microenvironment of responsive tumors

**Fig. 2:**
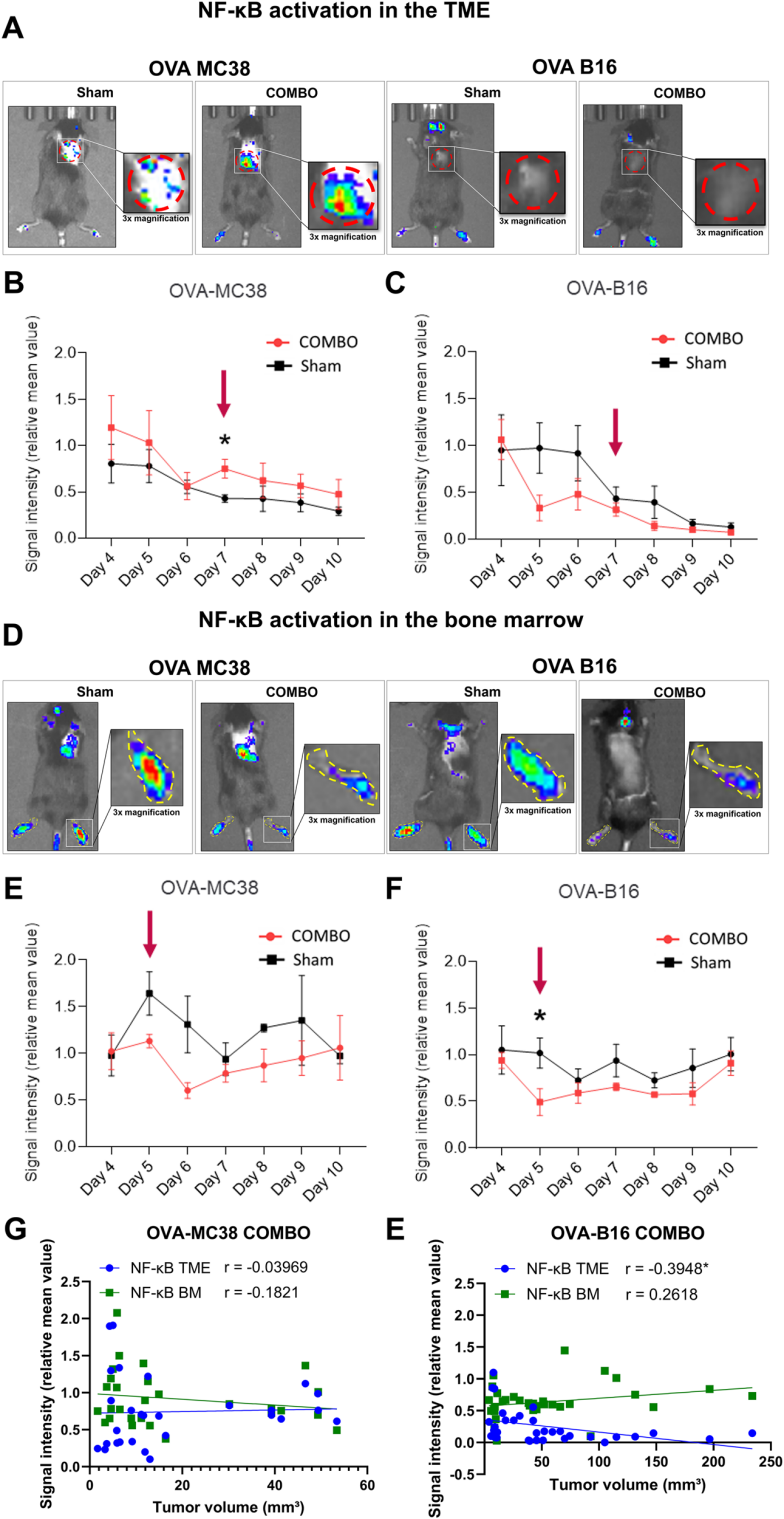
Monitoring of NF-κB activity (relative S.I.) in OVA-B16- and OVA-MC38-bearing ^NF-κB^Luc-reporter mice during COMBO or sham treatment using BLI. **A** Representative BLI images of OVA-MC38- and OVA-B16 tumor-bearing ^NF-κB^Luc-reporter mice treated with COMBO or sham. Representative images illustrate the radiance of the BLI signal in the tumor region on the right shoulder (indicated by a red dashed circle) on day 7 after tumor cell inoculation. The tumor area remained consistent across all the experimental mice. Graphs illustrate the relative SI of the BLI signal, representing NF-κB activation, in the TME of (**B**) OVA-B16 tumor-bearing and (**C**) OVA-MC38 tumor-bearing (day 7: *p = 0.0159) ^NF-κB^Luc reporter mice. **D** Representative images illustrate the radiance of the BLI signal in the hind limbs, representing the BM (outlined with a yellow dashed line) on day 5 after tumor cell inoculation. Furthermore, the graphs show the relative SI of the BLI signal of the BM of (**E**) OVA-B16 tumor-bearing (day 5: *p = 0.0410) and (**F**) OVA-MC38 tumor-bearing ^NF-κB^Luc reporter mice. For statistical evaluation, the SIs of the two experimental groups were compared on the same day using an unpaired parametric t test. *n* = 4–5. The average radiance (p/s/cm²/sr) of the SI can be found in Supplementary Fig. 2. **G** Correlation plots of tumor size and relative BLI signal intensity in the TME and BM over time (days 5–10) in COMBO-treated OVA-MC38 tumor-bearing mice. Pearson correlation coefficients (two-tailed). **E** Correlation plots of tumor size and relative BLI signal intensity in the TME and BM over time (days 5–10) in COMBO-treated OVA-B16 tumor-bearing mice. Pearson correlation coefficients (two-tailed).

In this study, we monitored NF-κB activation upon combined cancer immunotherapy in OVA-MC38 or OVA-B16 tumor-bearing ^NF-κB^Luc-reporter mice. We conducted bioluminescence optical imaging (BLI) from day 4 after tumor cell inoculation to noninvasively asses NF-κB activity in the tumor microenvironment (TME) and bone marrow (BM) of COMBO- and sham-treated mice. Representative BLIs of OVA-MC38 and OVA-B16 tumor-bearing ^NF-κB^Luc-reporter mice treated with COMBO or sham are shown in Fig. 2A.

COMBO treatment resulted in sustained NF-κB activation in the TME of OVA-MC38 tumors throughout the study (Fig. 2B). On day 4 following the onset of treatment, NF-κB activity in the TME of OVA-MC38 tumors was significantly greater in COMBO-treated mice (0.75 ± 0.10) than in the sham-treated controls (0.43 ± 0.14; *p* = 0.0159). In contrast, NF-κB activity in the TME of OVA-B16 tumors of COMBO-treated mice (1.06 ± 0.22) did not differ from that in the TME of sham-treated controls (0.95 ± 0.38; *p* = *0.0410*) (Fig. 2C). Our data suggest that COMBO treatment enhances NF-κB activation and consequently immune cell activation in the TME, as confirmed by the absolute SI values of the average radiance (p/s/cm²/sr) (Supplementary Fig. 2). Interestingly, all groups showed a decline in signal intensity (SI) over time, likely reflecting reduced signal detection with increasing tumor size.

### COMBO treatment reduced NF-κB activation in the bone marrow of sensitive and nonsensitive tumors

Numerous studies have shown that sufficient hematopoiesis is a prerequisite for successful cancer immunotherapy, as it promotes the egress of myeloid cells and induces progenitor cell proliferation within the BM.^28^ Additionally, hematopoiesis is tightly linked to NF-κB activation within the BM.^29^ Therefore, we focused on NF-κB activation patterns in the BM, which is a primary lymphoid organ. For the analysis of BM-associated NF-κB activation, we quantified the BLI signal from regions of interest (ROIs) encompassing the hind limbs, which reliably capture reporter activity originating from the BM compartment.^30^ The ankles of hind limbs were specifically selected to avoid the need for shaving and depilation, which can induce skin irritation and lead to nonspecific NF-κB activation. Interestingly, the results of the BLI analysis of the hind limbs of OVA-MC38 experimental mice revealed a reduction in NF-κB activation in the BM throughout the entire treatment period in COMBO-treated mice compared with that in their sham-treated littermates (Fig. 2D). Like in OVA-MC38 tumor-bearing mice, a significant decrease in NF-κB activity in the BM was observed in OVA-B16 tumor-bearing mice (Fig. 2E) one day after the administration of therapeutic OVA-T_H_1 cells (day 5).

To further assess the relationship between tumor progression and NF-κB activation, we performed an intra-individual correlation analysis between tumor size and NF-κB activity measured by BLI in the TME and BM over time (days 5–10) in COMBO-treated OVA-MC38 and OVA-B16 tumor-bearing mice. No consistent correlation was observed at the individual level in either compartment (Supplementary Fig. 9).

However, group-level analysis revealed distinct trends. In nonresponding OVA-B16 tumors, tumor size showed a weak positive trend with NF-κB activity in the BM (*r* = 0.26), although this did not reach statistical significance, whereas responding OVA-MC38 tumors exhibited a weak inverse relationship (*r* = −0.18) (Fig. 2 G and E). In the TME, NF-κB activity remained relatively stable in OVA-MC38 tumors (*r* = 0.04) but negatively correlated with tumor size in OVA-B16 tumors (r = −0.39, *p = 0.019) (Fig. 2 G and E).

### Histological and immunohistochemical evaluation of OVA-B16 and OVA-MC38 tumors

**Fig. 3:**
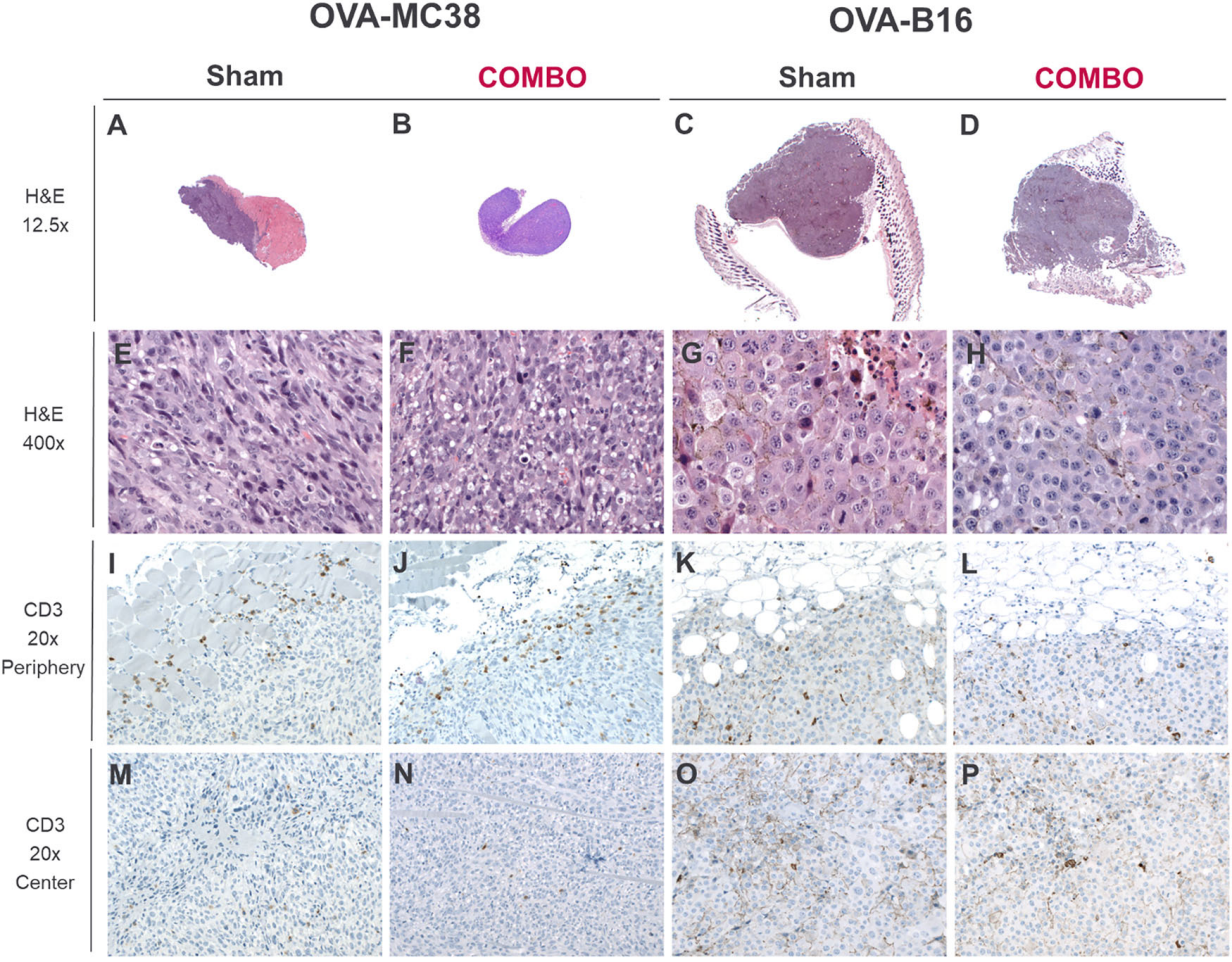
Histopathological (H&E) and IHC (CD3) analyses of OVA-MC38 and OVA-B16 tumors in ^NF-κB^Luc-reporter mice 7 days after the onset of COMBO or sham treatment. Representative H&E images of OVA-MC38 tumors (left) and OVA-B16 tumors (right) from both therapy groups are shown in overview (magnification: 12.5x; **A**–**D**) and in detail (magnification: 400x; **E**–**H**). Representative CD3 IHC of T cells from both experimental groups, showing the tumor margin (magnification: 20x; periphery; **I**–**L**) and the tumor center (magnification: 20x; center; M-P). *n* = 2–4.

To assess tumor morphology and the spatial distribution of immune cell infiltrates within the tumor microenvironment, we performed histological and immunohistochemical analyses of OVA-MC38 and OVA-B16 tumors. Histopathological analysis of the OVA-MC38 tumors in both treatment groups (COMBO; Sham) revealed relatively small tumors (Fig. 3A, B) composed of medium-sized spindle-shaped cells with polymorphic nuclei containing multiple large nucleoli (Fig. 3E, F). The inflammatory infiltrate was modest and predominantly localized at the tumor margin. Consistent with these findings, immunohistochemical staining revealed accumulation of CD3^+^ T cells mainly at the tumor margin, with only sparse infiltration into the tumor center (Fig. 3I, J). No obvious difference in T-cell distribution within the TME of OVA-M38 tumors was observed between the COMBO- and sham-treated tumors. Furthermore, we observed scattered B220^+^ B cells in the periphery of OVA-MC38 tumors, with only few cells present in the tumor center (green arrow, Supplementary Fig. 4).

In the OVA-B16 tumors from both treatment groups, high rates of mitosis and apoptosis were observed. The inflammatory infiltrate was low to mild at the tumor margins and minimal in the tumor center. Immunohistochemical evaluation of both therapy groups revealed increased accumulation of T cells (CD3^+^) in the tumor periphery (Fig. 3K, L) and lower T-cell numbers in the tumor center (Fig. 3O, P). Similarly, IHC analysis revealed greater numbers of B cells (B220) in the tumor periphery and fewer B cells in the center of OVA-B16 tumors (Supplementary Fig. 4) in COMBO-treated and sham-treated experimental mice. Notably, B-cell numbers in OVA-B16 tumors from COMBO-treated mice (OVA-T_H_1 + ICB) tended to be higher than those in tumors from isotype mAb-treated animals (Supplementary Fig. 4).

### COMBO treatment induces NF-κB activation in sensitive OVA-MC38 tumors

Noninvasive in vivo imaging of tumor-bearing NF-κBLuc reporter mice revealed NF-κB activation exclusively in COMBO-treated, therapy-sensitive OVA-MC38 tumors, but not in therapy-resistant OVA-B16 melanomas (Fig. 2). To identify the cellular source of this signal within the TME, we performed IF staining for luciferase, as a readout of NF-κB activation, together with immune cell markers.

**Fig. 4:**
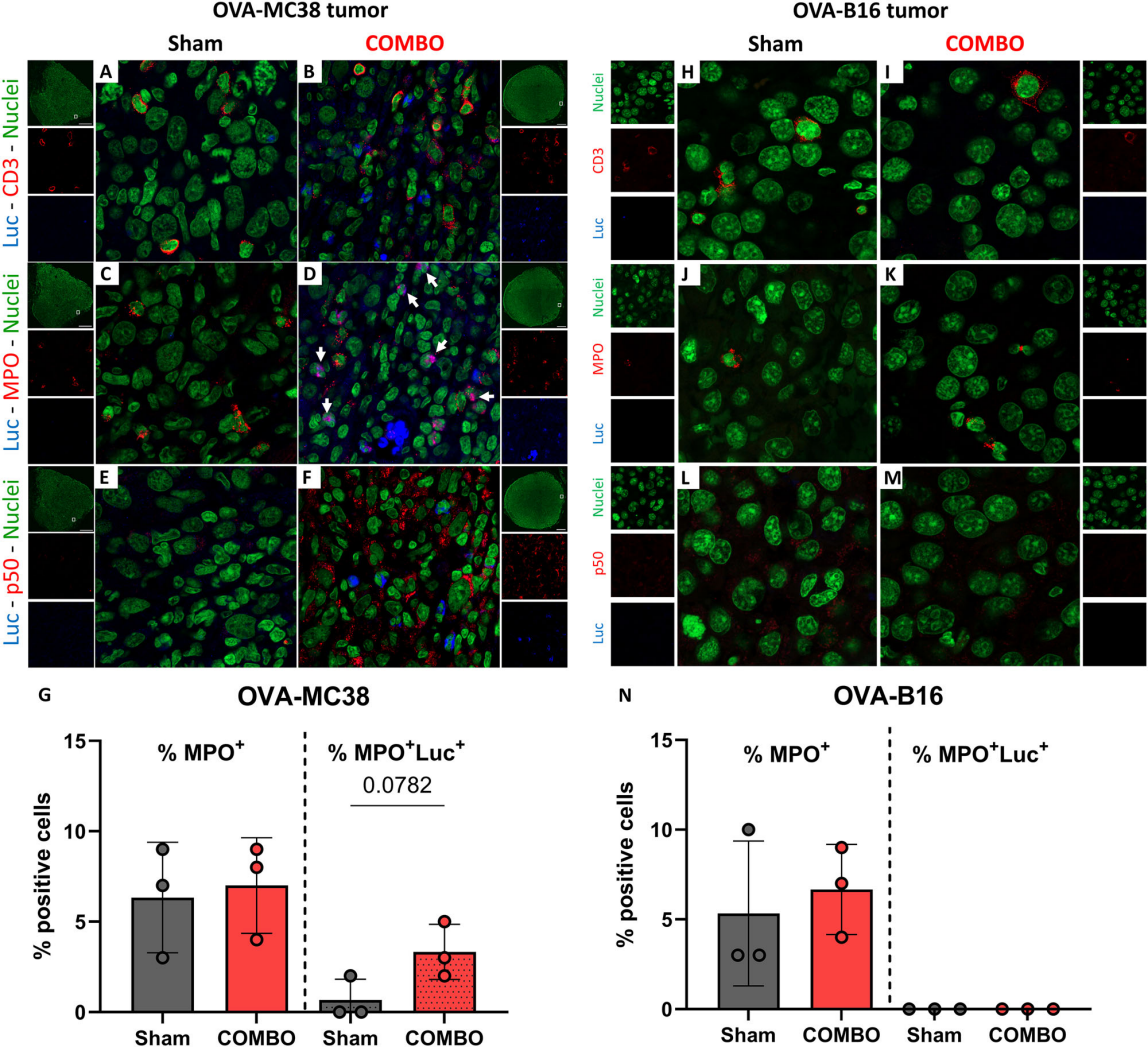
Immunofluorescence microscopy analysis of OVA-MC38 and OVA-B16 tumors from ^NF-κB^Luc-reporter mice that underwent COMBO or sham treatment. Seven days after the onset of treatment, OVA-MC38 and OVA-B16 tumors were harvested and analyzed by IFM for luciferase (blue), (**A**, **B**, **H**, **I**) CD3 (red), (**C**, **D**, **J**, **K**) MPO (red) and (**E**, **F**, **L**, **M**) p50 (red) expression. **G**, **N** Quantification of neutrophils (MPO + ) and luciferase positive neutrophils (MPO+Luc + ) in the TME of OVA-MC38 (**G**) and OVA-B16 (**N**) tumors. Statistical comparisons between groups were performed using unpaired t-tests with Welch’s correction. *n* = 3 per group.

Consistent with the in vivo imaging data, luciferase expression was readily detectable in the OVA-MC38 tumors from COMBO-treated mice but was largely absent in tumors from sham-treated animals (Fig. 4A–F), indicating treatment-induced NF-κB activation in host-derived cells. In contrast, no luciferase expression was detected in COMBO- or sham-treated OVA-B16 tumors (Fig. 4H-M), confirming the in vivo imaging results.

Immunofluorescence analysis identified scattered CD3⁺ T cells and MPO⁺ myeloid cells within the TME of both COMBO- or sham-treated OVA-MC38 and OVA-B16 tumors. However, costaining for CD3 and luciferase did not reveal luciferase positive T cells in any experimental group (Fig. 4A, B and H, I). In contrast, costaining for MPO and luciferase revealed luciferase-positive neutrophils (MPO⁺Luc⁺) within COMBO-treated OVA-MC38 tumors (Fig. 4D and G), indicating NF-κB activation predominantly in tumor infiltrating neutrophils. No luciferase-positive neutrophils were detected in OVA-B16 tumors under either COMBO- or sham-treatment (Fig. 4K and N).

These findings supporting the notion that COMBO therapy promotes NF-κB activation primarily in tumor-infiltrating neutrophils in the responsive OVA-MC38 tumors, whereas such activation is absent in nonresponsive OVA-B16 tumors.

To further characterize NF-κB pathway activation, we examined p50 expression. Increased nuclear and cytoplasmic p50 staining was detected in the TME of COMBO-treated OVA-MC38 tumors but not in tumors from sham-treated mice (Fig. 4E, F), consistent with activation of NF-κB signaling in the tumor microenvironment following COMBO therapy. In contrast, OVA-B16 tumors did not show increased p50 expression upon COMBO treatment compared with sham-treated controls (Fig. 4L, M), confirming their lack of responsiveness to immunotherapy.

### Investigation of the immune cell composition of tumors and lymphatic organs

**Fig. 5:**
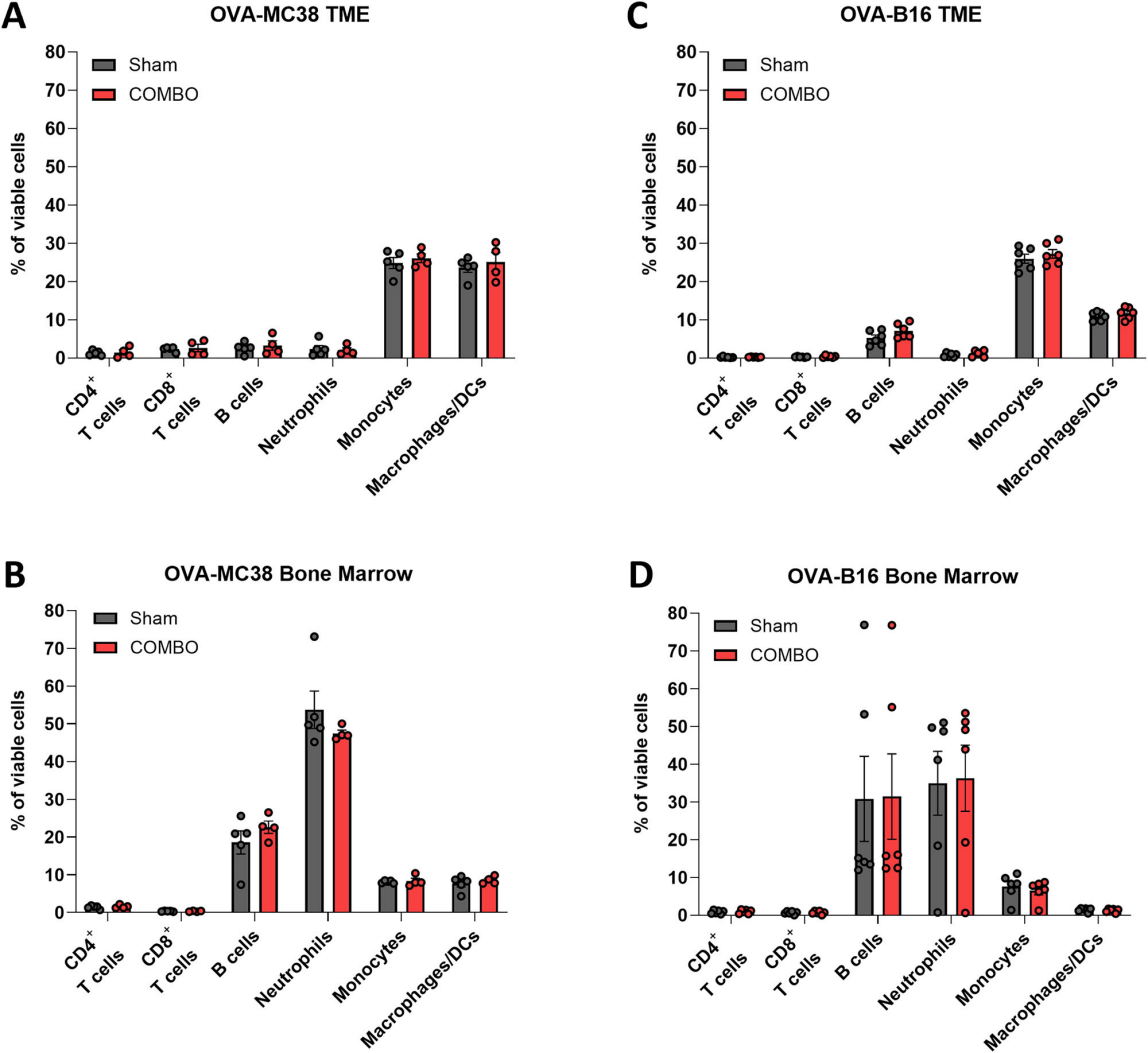
Flow cytometry analysis of tumor-infiltrating immune cells in OVA-MC38 and OVA-B16 tumors of ^NF-κB^Luc-reporter mice treated with COMBO or sham. Seven days after the onset of treatment, the percentages of CD4^+^ and CD8^+^ T cells, B cells (CD19^+^), neutrophils (CD11^+^Gr-1^high^), monocytes (CD11^+^Gr-1^low^), and macrophages/DCs (CD11^+^Gr-1^neg^) cells were determined in the tumors (**A**) and the BM (**B**) of OVA-MC38 tumor-bearing mice and tumors (**C**) and the BM (**D**) of OVA-B16 tumor-bearing mice. For statistical analysis of the flow cytometry data, a two-factor ANOVA with Šidák’s correction for multiple testing was used. A bar represents the mean ± SEM of each group. *n* = 4–6.

At the end of the experiment on day 10, after the last optical imaging measurement, experimental mice were euthanized, and half of the tumors as well as BM, spleens, draining lymph nodes, and control lymph nodes were collected and subjected to multicolor flow cytometry for quantitative characterization of immune cell populations and their relative distribution across tissues. First, the relative frequencies of CD4^+^ T cells, CD8^+^ T cells, CD19^+^ B cells, and neutrophils (CD11^+^Gr-1^high^), monocytes (CD11^+^Gr-1^low^), and macrophages/DCs (CD11^+^Gr-1^neg^) cells within the TME and in the BM as the primary lymphatic organ were determined. We detected comparable numbers of tumor-infiltrating CD4⁺ T cells in COMBO-treated (1.48 ± 1.36%) and sham-treated (1.31 ± 0.62%) OVA-MC38 tumor-bearing mice. Similarly, the percentage of CD8⁺ T cells did not significantly differ between the COMBO-treated (2.64 ± 1.91%) and sham-treated (2.34 ± 0.62%) groups (Fig. 5A). These findings are consistent with our IHC and IFM analyses, in which we did not observe COMBO-induced increases in CD3 T cells. Interestingly, a trend toward a reduced frequency of neutrophils was detected in the BM of COMBO-treated OVA-MC38 tumor-bearing mice (47.48 ± 1.74%) compared with that in the BM of sham-treated controls (53.74 ± 11.08%; *p* = 0.1522, Fig. 5B), which was not the case in the BM of nonresponsive OVA-B16 tumor-bearing mice (Fig. 5D). suggesting that COMBO treatment may induce the egress of neutrophils from the BM of responsive OVA-MC38 bearing mice.

## Discussion

In this study, we demonstrate that our novel COMBO treatment regimen, composed of adoptively transferred OVA-T_H_1 cells, anti-PD-L1 mAbs and anti-LAG-3 mAbs, effectively suppresses tumor growth in immunotherapy-responsive OVA-MC38 tumor-bearing ^NF-κB^Luc-reporter mice (Fig. 1A, B). In sharp contrast, immunotherapy-resistant OVA-B16 melanomas did not respond to COMBO treatment (Fig. 1C). These findings are consistent with those of previous studies demonstrating that immune checkpoint blockade enhances antitumor immunity primarily by promoting T-cell activation and proliferation.^31^ A key innovation of our work is the longitudinal, noninvasive in vivo monitoring of NF-κB activity using ^NF-κB^Luc-reporter mice. This approach, originally developed by Carlsen et al.^32^ allowed us to dynamically track NF-κB activation in endogenous immune and stromal cells within both the TME and the BM of responsive and nonresponsive tumor-bearing mice throughout the course of therapy (Fig. 2A-E). COMBO treatment induced NF-κB activation exclusively within the TME of responsive OVA-MC38 tumors. In striking contrast, COMBO treatment of OVA-B16 tumor-bearing mice failed to elicit any detectable NF-κB activity within the TME (Fig. 2A, C).

NF-κB is a central regulator of inflammatory and immune responses, and its activation is known to enhance antigen presentation and promote cytotoxic T-cell recruitment.^33^ Our findings suggest that COMBO-induced NF-κB activation is associated with a strong antitumoral immune response in OVA-MC38 tumor-bearing mice. In contrast, no NF-κB activation was detected in refractory OVA-B16 tumors under COMBO-treatment, suggesting a lack of effective immune activation in these tumors. Barnes et al. demonstrated that mice with impaired T-cell NF-κB activity failed to reject subcutaneous MC57-SIY tumors, whereas wild-type T cells successfully eliminated these tumors. These findings indicate that NF-κB activation within T cells is crucial for antitumor immunity, as it enables their functional maturation into effective cytokine-producing effector T cells.^34^

Interestingly, Liu et al. reported that adoptively transferred OVA-Th1 cells expressed high levels of PD-1 after they were transferred into tumor-bearing mice. Accordingly, the combination with anti-PD-L1 mAbs exhibited an additional antitumoral effect.^25^ More generally, Lim et al. found that immune cell-driven (e.g., by secretion of TNF and IFN-γ) NF-κB can regulate anticancer immune responses by increasing PD-L1 expression in tumor cells. Mechanistically, NF-κB not only promotes PD-L1 transcription but also contributes to the posttranslational stabilization of PD-L1 through ubiquitination.^35^ Importantly, although our COMBO therapy regimen, including blockade of the anti-PD-L1 mAbs, effectively disrupted the PD-L1–PD-1 axis, NF-κB activity within the TME of OVA-MC38 tumors remained elevated. Thus, persistent NF-κB activity within the TME might favor therapeutic efficacy.

The tumor models used in this study differ in intrinsic immunogenicity, with OVA-MC38 representing an immunogenic (“inflamed”) phenotype and OVA-B16 a poorly immunogenic (“non-inflamed”) phenotype.^36^ Accordingly, differences in NF-κB activation may reflect their distinct capacity to mount effective immune responses, indicating that NF-κB activity should be interpreted as a functional readout of therapy-induced immune engagement rather than a universal biomarker. Its broader applicability will require validation across additional tumor models.

Notably, all groups showed a decline in TME signal intensity over time, likely reflecting limitations of bioluminescence imaging in growing tumors, where increasing tumor size can reduce or decouple signal intensity from tumor burden due to optical attenuation and impaired substrate delivery.^37^

Furthermore, our study provides insight into the systemic immune response and immune cell dynamics by concurrently monitoring NF-κB activation in the BM in vivo. COMBO treatment resulted in decreased NF-κB activity in the BM of both OVA-MC38 and OVA-B16 tumor-bearing mice (Fig. 2D, E). In COMBO-treated OVA-MC38 tumor-bearing mice, this decrease was accompanied by a trend toward reduced frequencies of CD11b^+^Gr-1^high^ neutrophils in the BM, as determined by flow cytometry (Fig. 5B, D), suggesting therapy-induced mobilization of neutrophils from the BM into the periphery and infiltration into the TME. Consistent with this interpretation, our IFM analysis revealed abundant luciferase-positive neutrophils in COMBO-treated OVA-MC38 tumors, whereas neutrophils in sham-treated tumors were predominantly luciferase-negative (Fig. 4D, G). Notably, the overall frequency of neutrophils in the TME was similar between experimental groups, indicating phenotypic rather than quantitative differences. In contrast, COMBO-treated OVA-B16 tumors contained neutrophils lacking luciferase expression (Fig. 4K, N).

As luciferase expression reflects NF-κB activation, these findings indicate that COMBO treatment promotes the recruitment of NF-κB-active neutrophils into the TME of immunotherapy-responsive OVA-MC38 tumors. In contrast, neutrophils in nonresponsive OVA-B16 tumors remain largely NF-κB-inactive (Fig. 4 K, N). Together, these data suggest that NF-κB activation in tumor-infiltrating neutrophils may serve as a functional marker to distinguish responders from non-responders to combined immunotherapy.

Recent publications have shown that myeloid NF-κB activity acts as a double-edged sword in cancer immunity. In inflammation-driven tumorigenesis, myeloid NF-κB activation tends to promote tumor development.^18–20^ However, NF-κB activation can also drive immune activation in myeloid cells, which is needed for effective antitumor immunity and an optimal checkpoint response.^16,17^

Given that canonical NF-κB signaling plays an essential role in innate immunity and that p50 has been implicated in regulating neutrophil accumulation during inflammation,^38^ we next assessed p50 expression within the TME. Our IFM analyses demonstrated a pronounced induction of p50 expression in the TME of OVA-MC38 tumors (in both immune and tumor cells) from COMBO-treated mice. This pattern was absent in sham-treated OVA-MC38 tumors as well as in both COMBO- and sham-treated OVA-B16 tumors, suggesting that therapeutic efficacy is associated with enhanced NF-κB/p50 activity within the TME of responsive tumors (Fig. 5E, F and L, M). Proinflammatory stimulation, such as TNF stimulation, triggers canonical NF-κB activation in neutrophils, promoting p105 processing and thereby increasing nuclear p50 levels.^39^ Accordingly, the enhanced intratumoral p50 signal observed in our COMBO-treated, therapy-responsive tumors is consistent with the increased presence of NF-κB–active neutrophils. Notably, neutrophils are increasingly recognized for their dual role in cancer, as they can adopt distinct functional states that either promote or restrict tumor progression depending on the microenvironment.^40^ Linde et al. demonstrated that neutrophils are mobilized from the BM into the blood and infiltrate into the TME to successfully induce tumor cell regression upon immunotherapy. Specifically, they identified a therapy-induced recruitment of neutrophils to the TME via TNFR1 signaling and subsequent activation of the complement alternative pathway.^41^ In line with these findings, our data suggest that COMBO therapy drives neutrophils in immunotherapy-responsive OVA-MC38 tumors toward an activated, antitumor phenotype. This interpretation is supported by emerging evidence that distinct neutrophil subsets critically shape immunotherapy outcomes. For example, Gungabeesoon et al. showed that neutrophils switch to a proresponsive state during effective immunotherapy (anti-PD-L1 + anti-CTLA-4 mAbs) in KP lung tumor-bearing mice.^42^ Furthermore, recent studies have revealed that an IFN-γ-stimulated neutrophil subset characterized by increased Ly6E expression is correlated with an improved response to anti-PD-1 therapy.^43^

Our COMBO regimen has been previously characterized in RIP1Tag2 mice, where individual components (TA-Th1 cells, immune checkpoint inhibitors, and their combination) were extensively evaluated.^27^ In that study, TA-specific Th1 cells mediated the primary antitumor effect, which was further enhanced by immune checkpoint blockade, whereas ICIs alone showed limited efficacy. However, the specific contribution of each component to NF-κB activation cannot be determined from the present study and warrants further investigation.

Nevertheless, our findings indicate that increased NF-κB activation within the TME is associated with sensitivity to immunotherapy in this preclinical model. Thus, NF-κB activity may serve as a functional readout of therapy-induced immune activation, with potential relevance for response monitoring in preclinical and future translational studies.

Importantly, our NF-κB BLI approach enables noninvasive in vivo monitoring of stromal- and immune cell-derived NF-κB activity in the TME and BM of a cancer immunotherapy-responsive tumor model, providing a powerful tool for preclinical evaluation of therapeutic strategies that modulate immune activation.

In this context, longitudinal assessment of NF-κB activity during treatment may allow early discrimination between therapy-responsive and -resistant tumors. In our model, increased NF-κB activation within the TME during therapy was associated with treatment efficacy, whereas the absence indicated treatment resistance. Thus, NF-κB imaging could serve as an early functional readout of therapy-induced immune activation in preclinical studies.

While direct clinical translation would require the development of suitable imaging probes and standardized protocols, these findings support the concept that functional imaging of immune activation—rather than tumor burden alone—may improve assessment of antitumoral immune responses. Future studies are indispensable to determine whether NF-κB–based imaging approaches can be integrated for monitoring of cancer immunotherapies in translational and clinical settings.

## Methods

### Experimental mice

Female C6.CBA tg(3x-NF-κB-Luc) (^NF-κB^Luc reporter mice) were kindly provided by Harald Carlsen (Norwegian University of Life Sciences, Ås, Norway). These mice carry a transgene with 3 NF-κB-responsive elements and a modified firefly luciferase cDNA.^32^ C6.CBA tg(3x-NF-κB-Luc) mice were backcrossed to the C57BL/6 J genetic background for more than 10 generations. Female animals aged 8–10 weeks were kept in individually ventilated cages with standard rodent pellet food and water available ad libitum. Mice were allowed to acclimatize for at least 7 days before experiments. All animal experiments were performed at the University of Tübingen and approved by the local authorities (Regierungspräsidium Tübingen, approval number: R 1/16).

### Cell lines and reagents

OVA-MC38 tumor cells and OVA-B16 melanoma cells were cultured in DMEM (PAN Biotech, Aidenbach, Germany) supplemented with 10% fetal bovine serum (FBS; L-glutamine; Sigma‒Aldrich, St. Louis, MO, USA) and 100 U ml^-1^ penicillin/streptomycin (Biochrom, Berlin, Germany). OVA-MC38 adenocarcinoma cells were a gift from Dr. Danielle Arnold-Schild (University of Mainz, Institute for Immunology). The OVA-MC38 tumor cell culture medium was supplemented with G418 Geneticin (Life Technologies Limited, Paisley, UK).

### Experimental exogenous tumor models

Prior to tumor inoculation, the right shoulder of each animal was shaved with an electric razor (Wahl Pocket Pro Trimmer, Nobby, Bocholt, Germany), and the remaining hair was removed with depilatory cream (Veet, PZN: 07768307, Heidelberg, Germany) to prevent signal loss caused by the black fur. OVA-MC38 tumor cells (0.5 × 10^6^) in 100 µl of PBS were inoculated by *s.c*. injection into the right shoulder. OVA-B16 tumor cells (7.5 × 10^6^) were intracutaneously (orthotopic tumor model*)* injected into the right shoulder (orthotopic) in 25 µl of PBS. Tumor growth was monitored by determining two dimensions (length and width) with a caliper. The tumor volume was calculated using the formula ${Tumor\; volume}=\frac{{Length}* {{Width}}^{2}}{2}$.

### Combinatory cancer immunotherapy

Three days after tumor cell inoculation, the experimental mice as well as control mice (sham) were subjected to low-dose 2 Gy whole-body irradiation to deplete endogenous immune cells.^44^ Twenty-four hours later (four days after tumor cell inoculation), the experimental mice received an intraperitoneal (*i.p.)* injection of 10^7^ OVA-specific IFN-γ-producing CD4^+^ T cells (OVA-T_H_1) suspended in 200 µl of PBS. Sham-treated control mice were injected with 200 µl of PBS. Twenty-four hours later (five days after tumor cell inoculation), all mice received 200 µg of anti-PD-L1 mAbs (clone 10 F.9G2; BioXcell, West Lebanon, USA) and 200 µg of anti-LAG-3 mAbs (clone C9B7W; BioXcell) *i.p*. Sham-treated control mice were injected with IgG2b isotype mAbs (clone LTF-2; BioXcell) and IgG1 isotype mAbs (clone HRPN; BioXcell). ICI or isotype control mAb treatment was repeated on day seven and day nine after tumor cell inoculation. On day 10, all the mice were sacrificed, and the tumors and lymphatic organs of interest were isolated for histological, immunohistochemical (IHC) and multicolor flow cytometric analysis.

### In vivo bioluminescence optical imaging (BLI)

For semiquantitative noninvasive in vivo imaging of NF-κB activation, a dedicated preclinical optical imaging system (IVIS^®^ Spectrum, PerkinElmer, Rodgau-Jügesheim, Germany) was used. The mice were injected *i.p*. with 75 mg/kg luciferin (diluted in 100 µl of PBS) 5 min before the BLI measurement. Experimental mice underwent isoflurane anesthesia (1.5–2.0% in 100% oxygen) and placed on a heated platform in the imaging chamber. To assess the signal intensity (SI) in regions of interest (ROIs), tumors located at the right shoulder of experimental mice were uniformly delineated by circular regions in each image. The hind limbs were outlined to evaluate the SI in the BM.^30^ Analysis was performed using Living Image software (Perkin Elmer, Waltham, USA); SI values were obtained as the average radiance (p/s/cm²/sr). We conducted BLI from day 4 (after tumor cell inoculation) to noninvasively measure NF-κB activity in the TME and BM of COMBO-treated and sham-treated tumor-bearing ^NF-κB^Luc-reporter mice. To normalize the SI in the TME and BM for each experimental group, we determined the mean SI on day 4 and set it as the reference value of 1. For the subsequent days, we calculated the SI ratio by dividing the SI value of the respective day by the SI value on day 4 (p/s/cm²/sr). Representative images of tumor-bearing ^NF-κB^Luc-reporter mice from the two therapy groups are shown in Supplementary Fig. 1. After the final imaging measurements (day 10 after tumor cell inoculation), mice were deeply anesthetized with isoflurane (5% in oxygen) and euthanized by cervical dislocation to ensure death.

### Histology and immunohistochemistry

OVA-B16 melanomas and OVA-MC38 adenocarcinomas were isolated and cut in half, with one half fixed in 4% formalin and subsequently embedded in paraffin, while the other half was used for flow cytometry analysis. For histological evaluation, the tissue samples were cut into 3–5 µm sections and stained with hematoxylin and eosin (H&E). Immunohistochemistry was performed on an automated immunostainer (Ventana Medical Systems, Inc.) according to the company’s protocols for open procedures with slight modifications using mAbs against CD3 (SP7; DCS Innovative Diagnostik-Systeme GmbH & Co. KG) and B220 (Clone RA3-6B2; BD Biosciences, Becton, Dickinson and Company, Franklin Lakes, New Jersey, USA). The appropriate positive and negative controls were used to confirm adequate staining. The histologic samples were analyzed by an experienced pathologist (L. Quintanilla-Martinez). Photomicrographic images were acquired with an Axioskop 2 *plus* Zeiss microscope equipped with a Jenoptik (Laser Optik System, Jena, Germany) ProgRes C10 *plus* camera and software. Final image preparation was performed with Adobe Photoshop CS2.

### Immunofluorescence microscopy (IFM)

First, 5-µm-thick cryosections were fixed using periodate-lysine-paraformaldehyde, subsequently washed in PBS for 5 min and incubated in wash buffer for 5–10 min. Nonspecific binding sites were blocked with donkey serum (Sigma‒Aldrich) diluted 1:20. Next, the cryosections were incubated overnight with diluted solutions of the specific primary antibodies, followed by three washes in wash buffer. After being washed, the cryosections were incubated at room temperature with a diluted solution of the secondary antibody. This step was followed by three additional washes. Nuclear staining was performed by incubating the cryosections for 5 min at room temperature in YOPRO solution (Molecular Probe-Y 3603) diluted 1:2000 in PBS, followed by a 5 min wash in PBS. Finally, the sections were mounted on glass slides and analyzed using a confocal laser scanning microscope (LSM 800; Carl Zeiss Microscopy GmbH, Jena, Germany) with ZEN 2.3 software (Carl Zeiss). For quantitative analysis, MPO⁺ cells and MPO⁺Luc⁺ double-positive cells were quantified and normalized to the total number of nuclei per field. Image analysis was performed using ZEN 2.3 software, and multiple fields per tumor section were evaluated across tumors (*n* = 3 per group).

### Multicolor flow cytometry

OVA-B16 melanoma and OVA-MC38 adenocarcinoma tumor samples were passed through a 70 µm cell strainer and washed in PBS supplemented with 1% FBS. The cell suspensions, including tumor and immune cells (of the inflammatory TME), were counted with a C-Chip Disposable Counting Chamber (NanoEnTek, Seoul, Korea), and 5 × 10^6^ cells per sample were used for staining. We used the following mAbs for staining: 7AAD-Viability, APC-CD4, APC-Cy7-CD8, Pacific Blue-CD19, FITC-CD69 and FC-Block (all from BD Biosciences). The cell suspensions were analyzed on an LSR II flow cytometer (BD Biosciences) using FlowJo software (FlowJo, Ashland, USA).

### Statistical analysis

Experiments were not randomized, and investigators were not blinded to group allocation during experiments or outcome assessment. Group sizes ranged from *n* = 4–6 mice per group, as indicated in the figure legends and Results. Animals in which tumors failed to establish were excluded from analysis. No formal sample size calculation was performed; group sizes were chosen based on previous experience with similar experimental models.

For statistical evaluation, we used GraphPad Prism software (version 9.3.1; GraphPad Software, Inc., USA). We calculated the mean value and the standard error of the mean (SEM). The sample size (*n*) is provided in the respective figure legend. Statistical significance was determined using an unpaired Student t-test for each time point. Comparisons between relative tumor growth were performed using 2-way ANOVA and Sidak’s multiple comparison test. No animals were excluded from the reported analyses.

## Supplementary information

MOESM1_ESM.pdf

## Data Availability

The data supporting the findings of this study are available within the article and its Supplementary Information. Additional data are available from the corresponding author upon reasonable request.
