# Supplementary material for: Noninvasive in vivo imaging of NF-κB activation predicts immunotherapy response in solid tumors
Source: Npj Imaging. 2026 May 20;4:38. doi: 10.1038/s44303-026-00173-8 (PMC13230985; doi:10.1038/s44303-026-00173-8)
Supplement: Supplementary file 1 — MOESM1_ESM.pdf [file 44303_2026_173_MOESM1_ESM.pdf]

Supplemental Figure 1

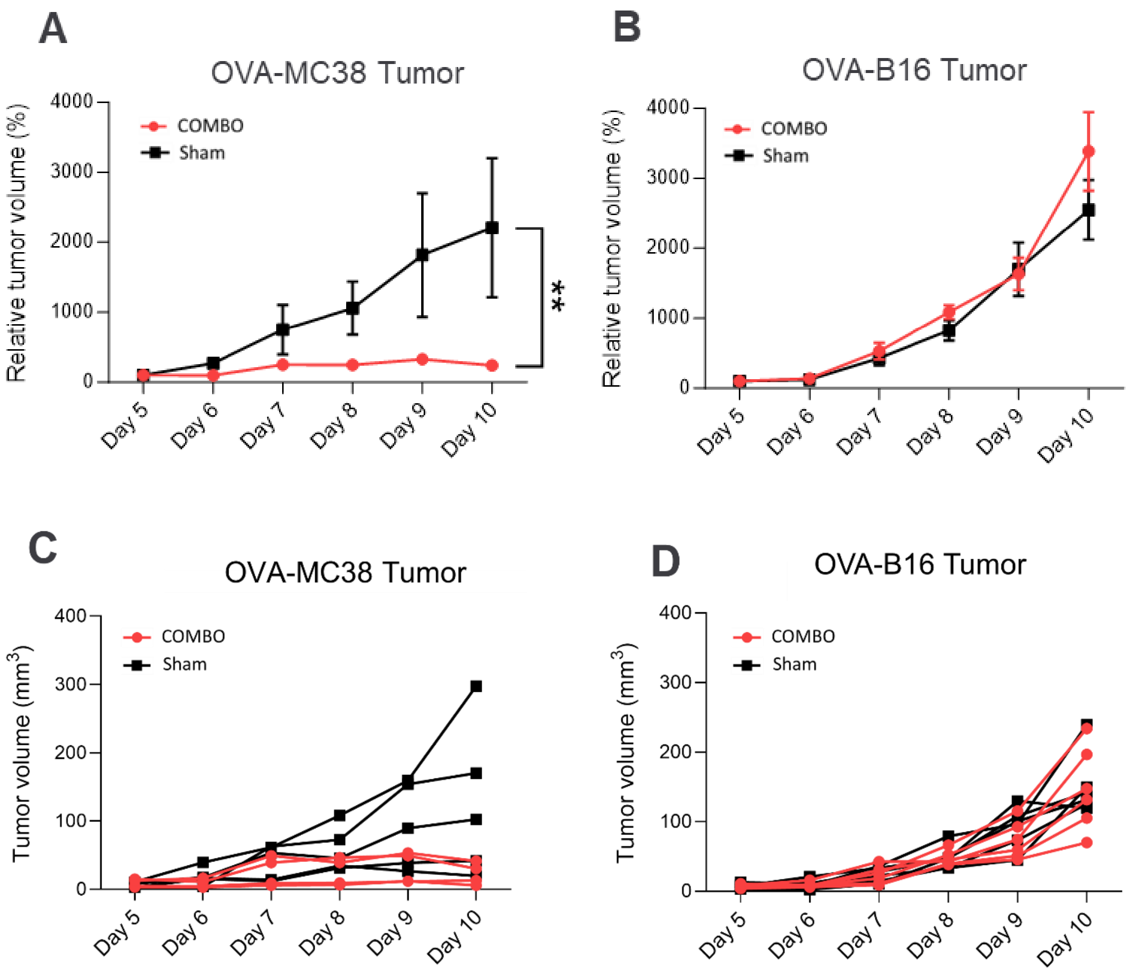

Supplemental Figure 1: COMBO treatment suppresses tumor growth in therapy-responsive OVA-MC38 tumors. All the mice were sacrificed ten days after tumor inoculation. From day 5 after tumor inoculation, the relative tumor volume of **(A)** OVA-MC38 and **(B)** OVA-B16 tumors was determined daily using calipers until the end of the trial on day 10. From day 5, the tumors were measurable in all experimental mice, which is why the tumor volumes on this day served as a reference value. The dots represent the mean ± SEM of one group: OVA-MC38 tumors on day 10. Comparisons between relative tumor growth were performed using 2-way ANOVA and Sidak's multiple comparison test (**p = 0.0289). n = 4–6. Individual values of the tumor volume (mm^3) are given for **(C)** OVA-MC38 and **(D)** OVA-B16 tumors.

Supplemental Figure 2

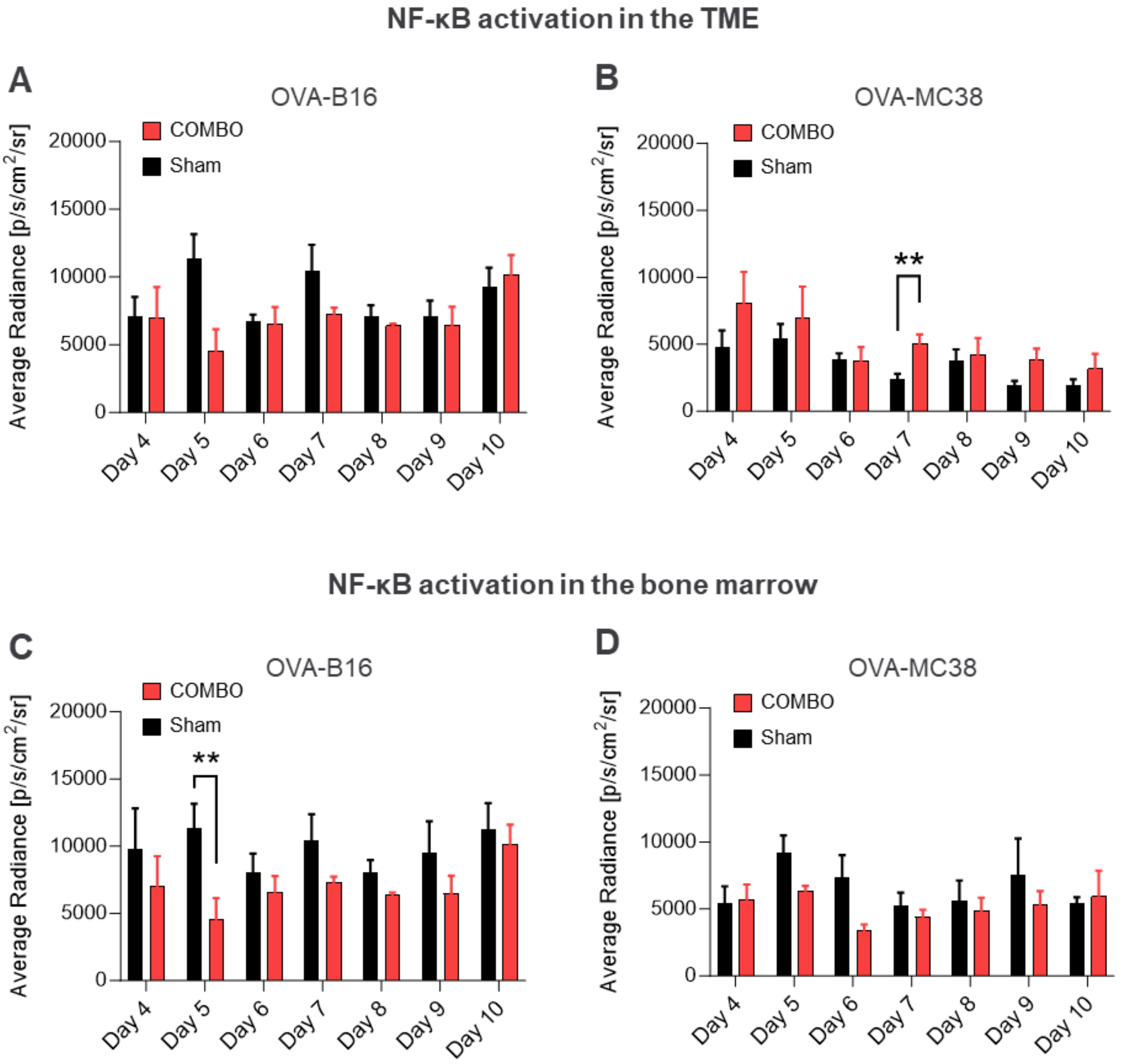

Supplemental Figure 2: Monitoring of NF-κB activity in OVA-B16- and OVA-MC38-bearing $^{NF\text{-}\kappa B}$Luc-reporter mice during treatment with COMBO or sham using BLI. $^{NF\text{-}\kappa B}$Luc-reporter mice were inoculated with *s.c.* OVA-B16 or OVA-MC38 tumor cells on day 0. After three days, the mice underwent initial 2 Gy low-dose whole-body irradiation. One day later (day 4), the experimental animals received an adoptive transfer of OVA-T_H1 cells or a PBS application (*i.p.).* On days 5, 7 and 9, *i.p.* the immune checkpoint inhibitors anti-PD-L1 and anti-LAG-3 (ICB) were administered intravenously. The graphs illustrate the average radiance in p/s/cm²/sr of the BLI-SI of the TME of **(A)** OVA-B16 tumor-bearing and **(B)** OVA-MC38 tumor-bearing (day 7: *p = 0.0092) $^{NF\text{-}\kappa B}$Luc reporter mice. Furthermore, the graphs show the average radiance in p/s/cm²/sr of the BLI-SI of the BM of **(C)** OVA-B16 tumor-bearing (day 5: *p = 0.0183) and **(D)** OVA-MC38 tumor-bearing $^{NF\text{-}\kappa B}$Luc reporter mice. For statistical evaluation, the two experimental groups were compared on the same day using an unpaired parametric t test. n = 4-6.

Supplemental Figure 3

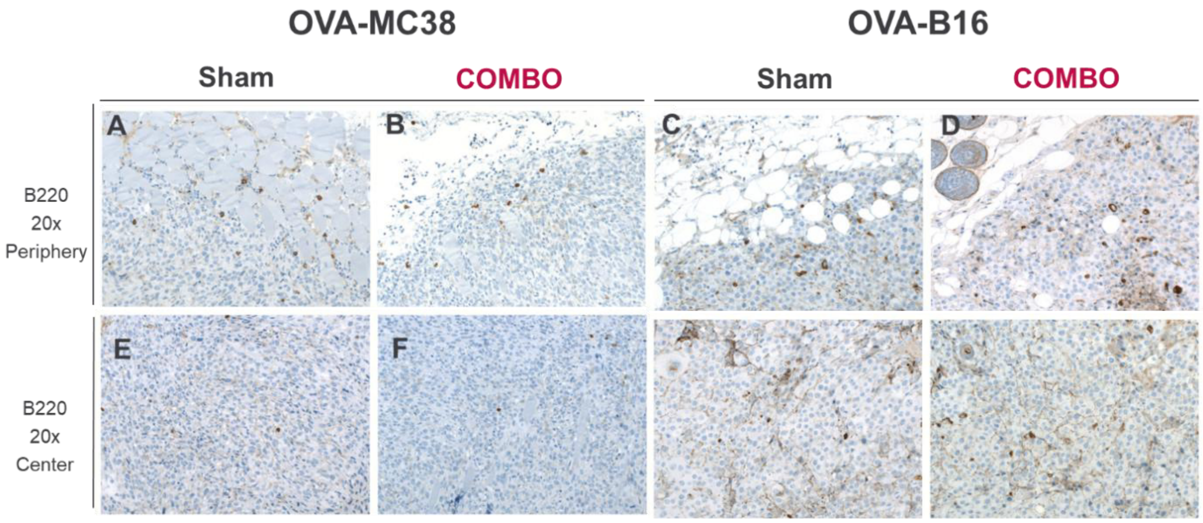

Supplemental Figure 3: Immunohistochemical (B220) analysis of the OVA-MC38 and OVA-B16 tumors of $^{NF\text{-}\kappa B}$Luc-reporter mice 10 days after tumor inoculation and treatment with COMBO or sham. Representative B220 immunohistochemistry of the B cells of the two experimental groups showing the marginal area of the tumor (magnification: 20x; periphery; **A-D**) and the tumor center (magnification: 20x; center; **E-H**). n = 4–6.

Supplemental Figure 4

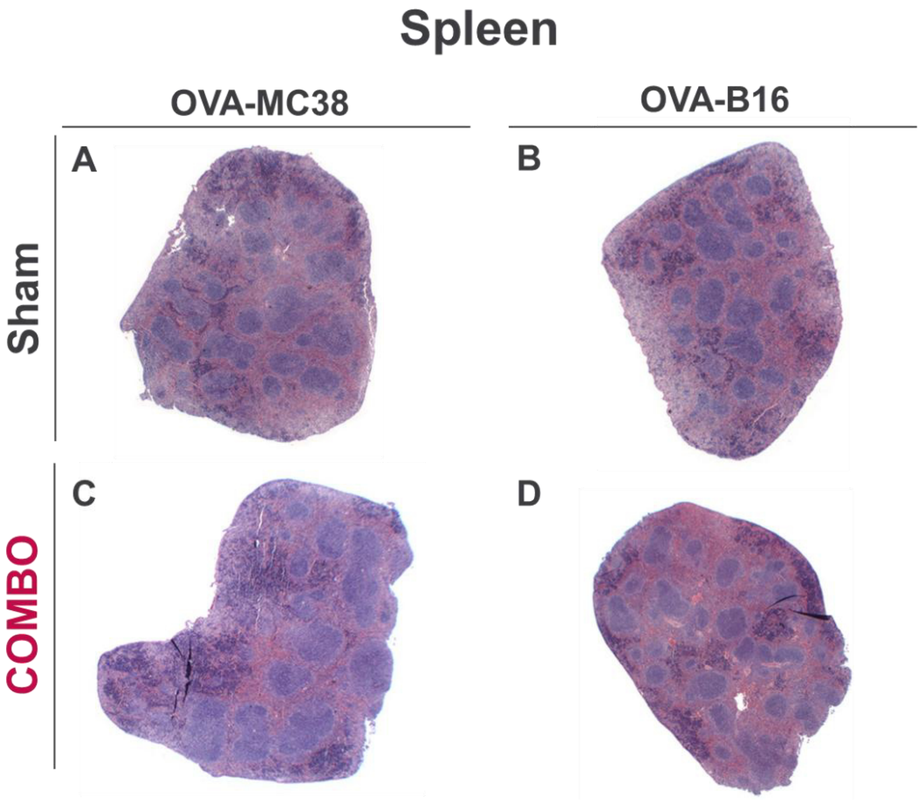

Supplemental Figure 4: Histopathological (H&E) analysis of the spleens of OVA-MC38 and OVA-B16 tumor-bearing $^{NF\text{-}\kappa B}$Luc-reporter mice 10 days after tumor inoculation and treatment with COMBO or sham. Representative H&E images of the spleens of OVA-MC38 (**A+C**) and OVA-B16 tumor-bearing $^{NF\text{-}\kappa B}$Luc-reporter mice (**B+D**) in both therapy groups are shown in the overview (magnification: 12.5x). n = 4–6.

Supplemental Figure 5

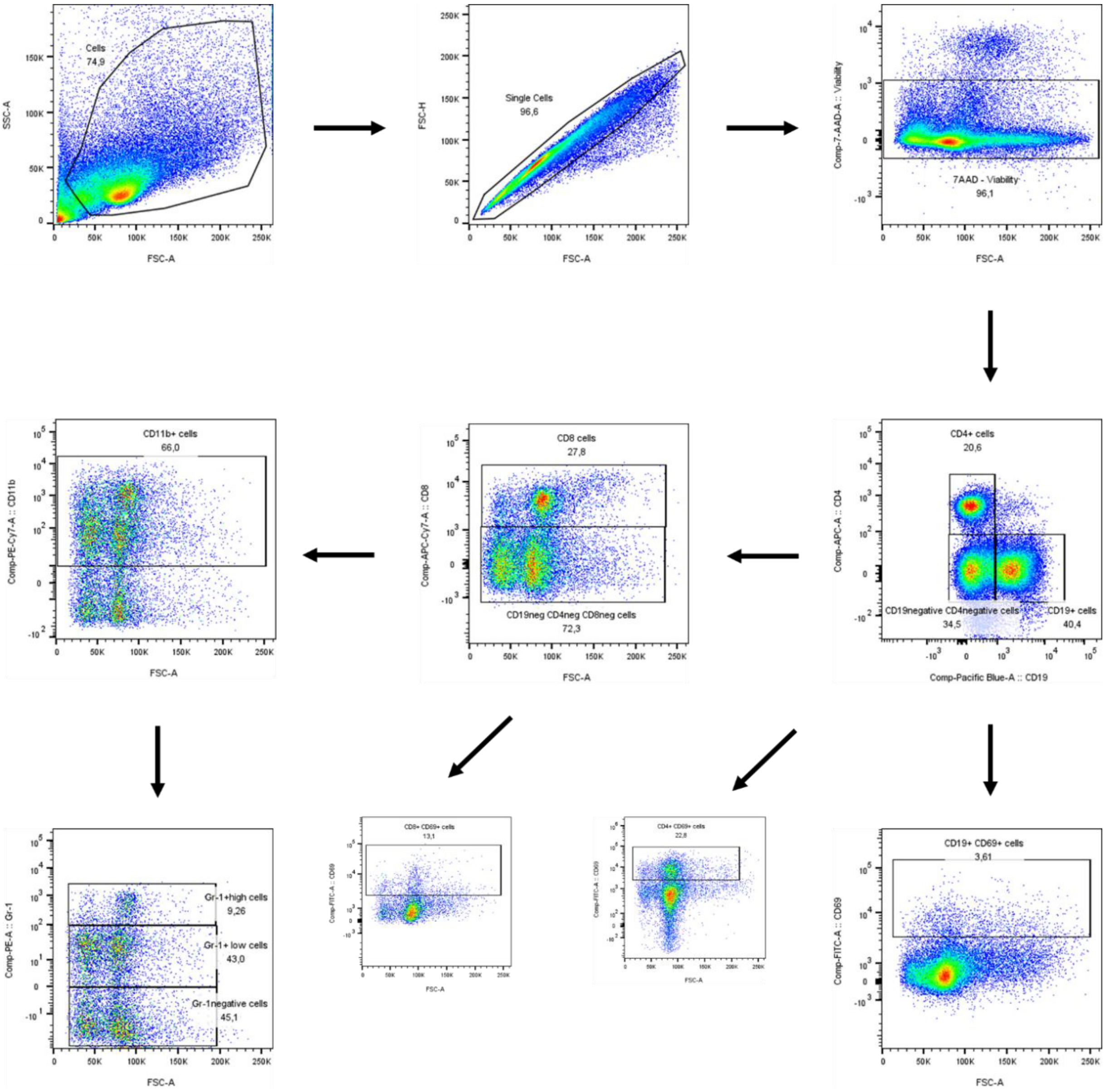

Supplemental Figure 5: Gating strategy for flow cytometric analysis of immune cell subsets. Forward scatter (FSC-A) and side scatter (SSC-A) were used to identify the lymphocyte population. Singlets were gated on FSC-A versus FSC-H to exclude doublets. Viable cells were identified by excluding 7-AAD-positive cells. CD19-negative and CD4-negative cells were selected to further gate $CD8^+$ T-cell subsets. $CD8^+$ and $CD4^+$ T cells and $CD19^+$ cells were subdivided into $CD8^+CD69^+$ activated T cells, $CD8^+CD69^+$ T cells and $CD19^+CD69^+$ cells. Furthermore, $CD11b^+$ cells were chosen to divide Gr-1 into Gr-1high, Gr-1low and Gr-1-negative cells. Data were acquired using a flow cytometer and analyzed using FlowJo software. Compensation controls were applied to ensure accurate gating.

Supplemental Figure 6

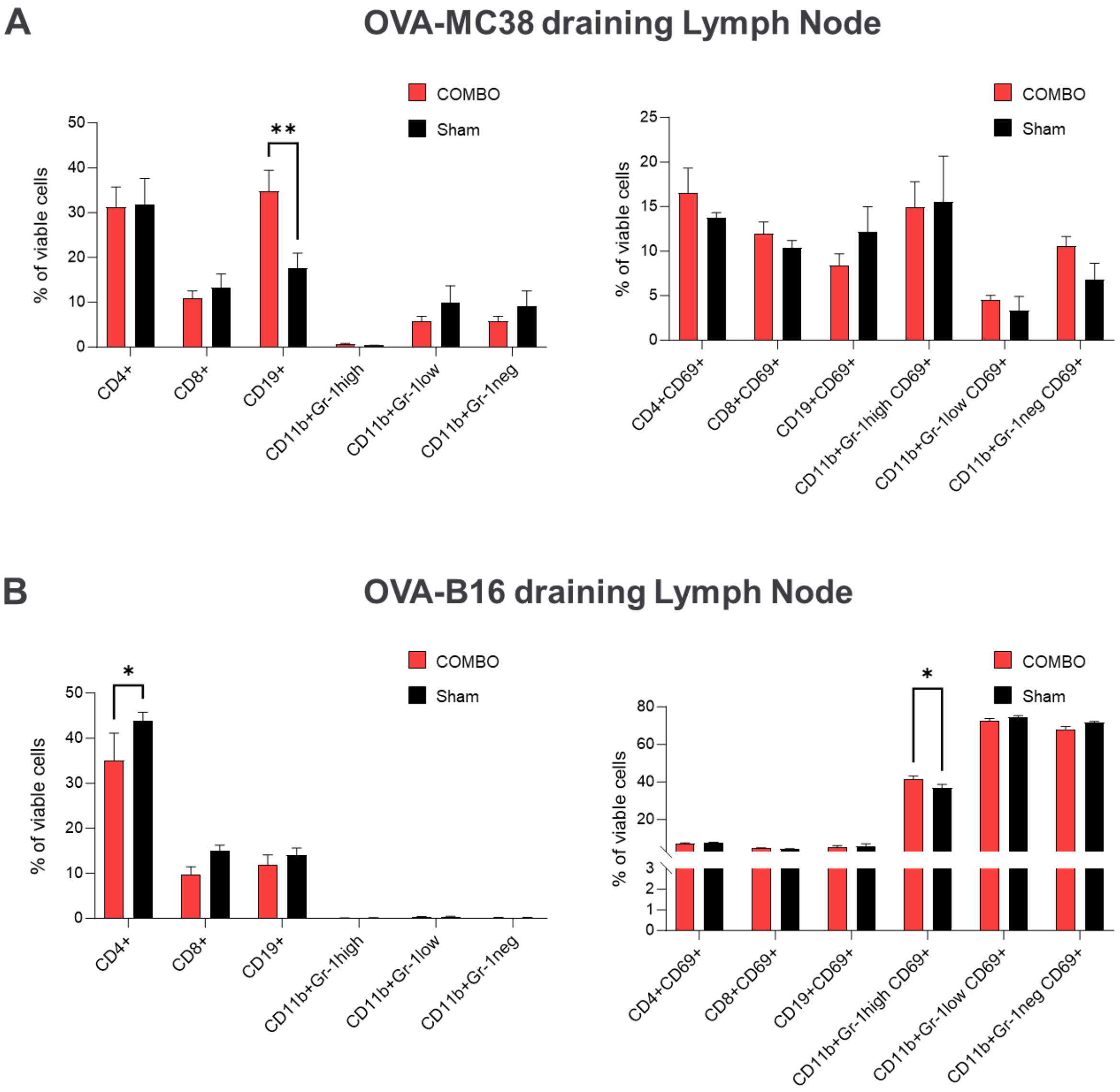

Supplemental Figure 6: Flow cytometry analysis of the percentage of immune cells in the draining lymph nodes of OVA-MC38 and OVA-B16 tumor-bearing $^{NF\text{-}\kappa B}$Luc-reporter mice treated with COMBO or sham. Ten days after tumor inoculation (7 days after therapy initiation), the percentages of CD4$^+$ and CD8$^+$ T cells, CD19$^+$ B cells, and CD11$^+$Gr-1high, CD11$^+$Gr-1low, and CD11$^+$Gr-1neg cells were determined in the **(A)** draining lymph nodes of OVA-MC38 and **(B)** BM of OVA-B16 tumor-bearing mice (left graph). In addition, the surface membrane expression of CD69 was investigated in all immune cells (right graph). For statistical analysis of the flow cytometry data, a two-factor ANOVA with Šidák's correction for multiple testing was used. A bar represents the mean ± SEM of each group. n = 4–6.

Supplemental Figure 7

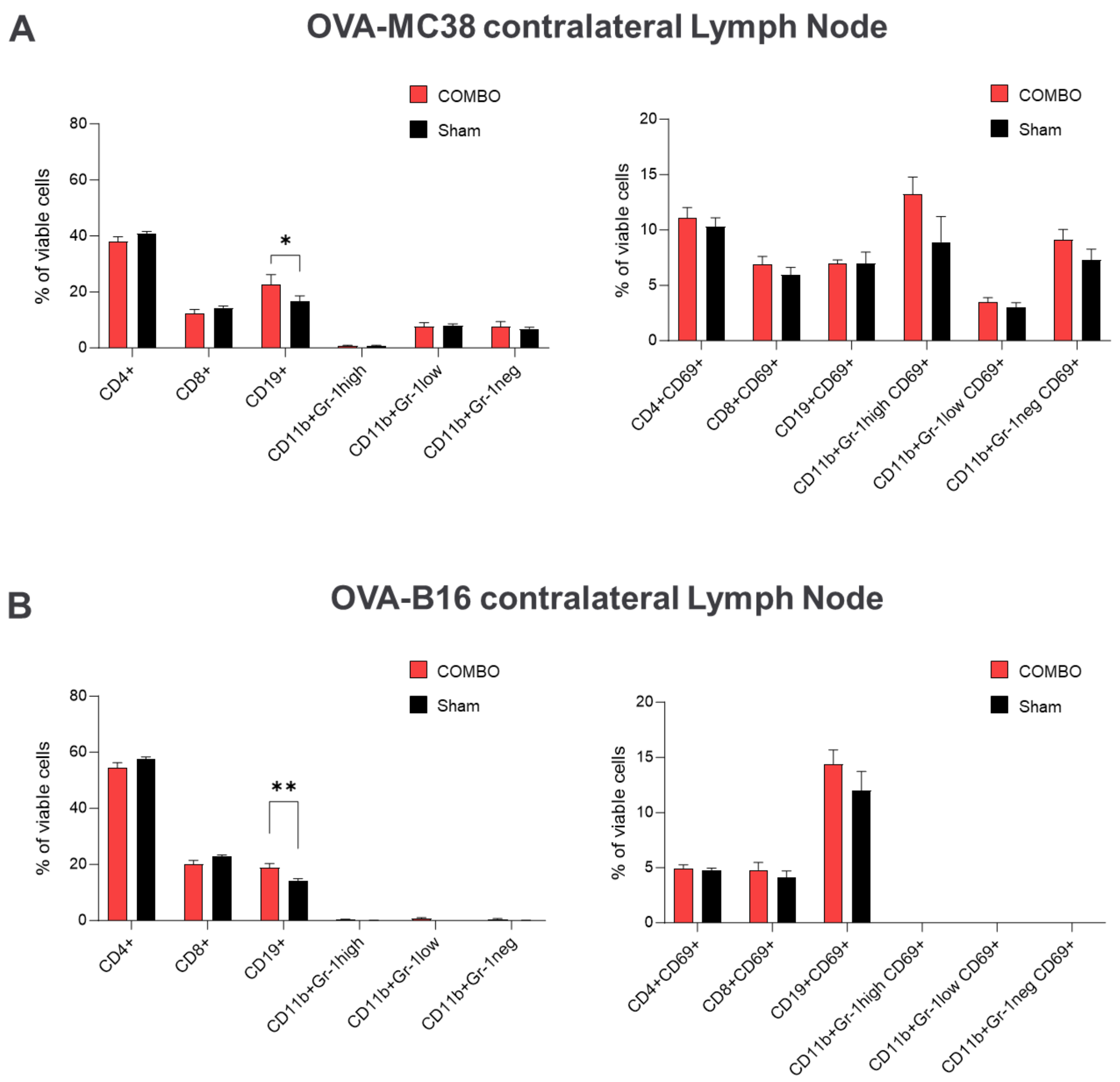

Supplemental Figure 7: Flow cytometry analysis of the percentage of immune cells in the contralateral lymph nodes of OVA-MC38 and OVA-B16 tumor-bearing $^{NF\text{-}\kappa B}$Luc-reporter mice treated with COMBO or sham. Ten days after tumor inoculation (7 days after therapy initiation), the percentages of CD4$^+$ and CD8$^+$ T cells, CD19$^+$ B cells, and CD11$^+$Gr-1high, CD11$^+$Gr-1low, and CD11$^+$Gr-1neg cells were determined in the **(A)** contralateral lymph nodes of OVA-MC38 and **(B)** BM of OVA-B16 tumor-bearing mice (left graph). In addition, the surface membrane expression of CD69 was investigated in all immune cells (right graph). For statistical analysis of the flow cytometry data, a two-factor ANOVA with Šidák’s correction for multiple testing was used. A bar represents the mean ± SEM of each group. n = 4–6.

Supplemental Figure 8

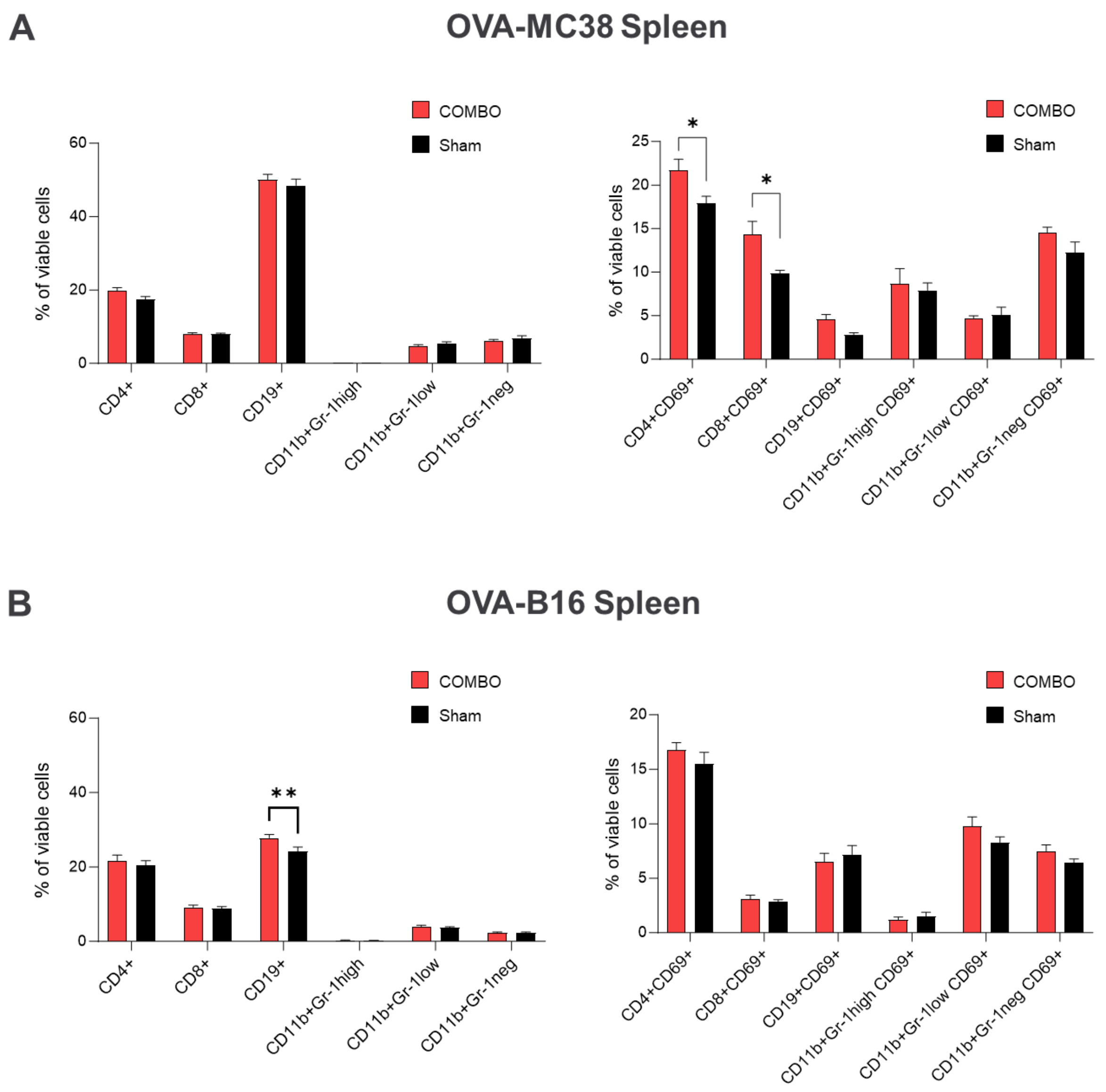

Supplemental Figure 8: Flow cytometry analysis of the percentage of immune cells in the spleens of OVA-MC38 and OVA-B16 tumor-bearing $^{NF\text{-}\kappa B}$Luc-reporter mice treated with COMBO or sham. Ten days after tumor inoculation (7 days after therapy initiation), the percentages of $CD4^+$ and $CD8^+$ T cells, $CD19^+$ B cells, and $CD11^+$Gr-1high, $CD11^+$Gr-1low, and $CD11^+$Gr-1neg cells were determined in the **(A)** spleens of OVA-MC38 and **(B)** BM of OVA-B16 tumor-bearing mice (left graph). In addition, the surface membrane expression of CD69 was investigated in all immune cells (right graph). For statistical analysis of the flow cytometry data, a two-factor ANOVA with Šidák’s correction for multiple testing was used. A bar represents the mean ± SEM of each group. n = 4–6.

Supplemental Figure 9

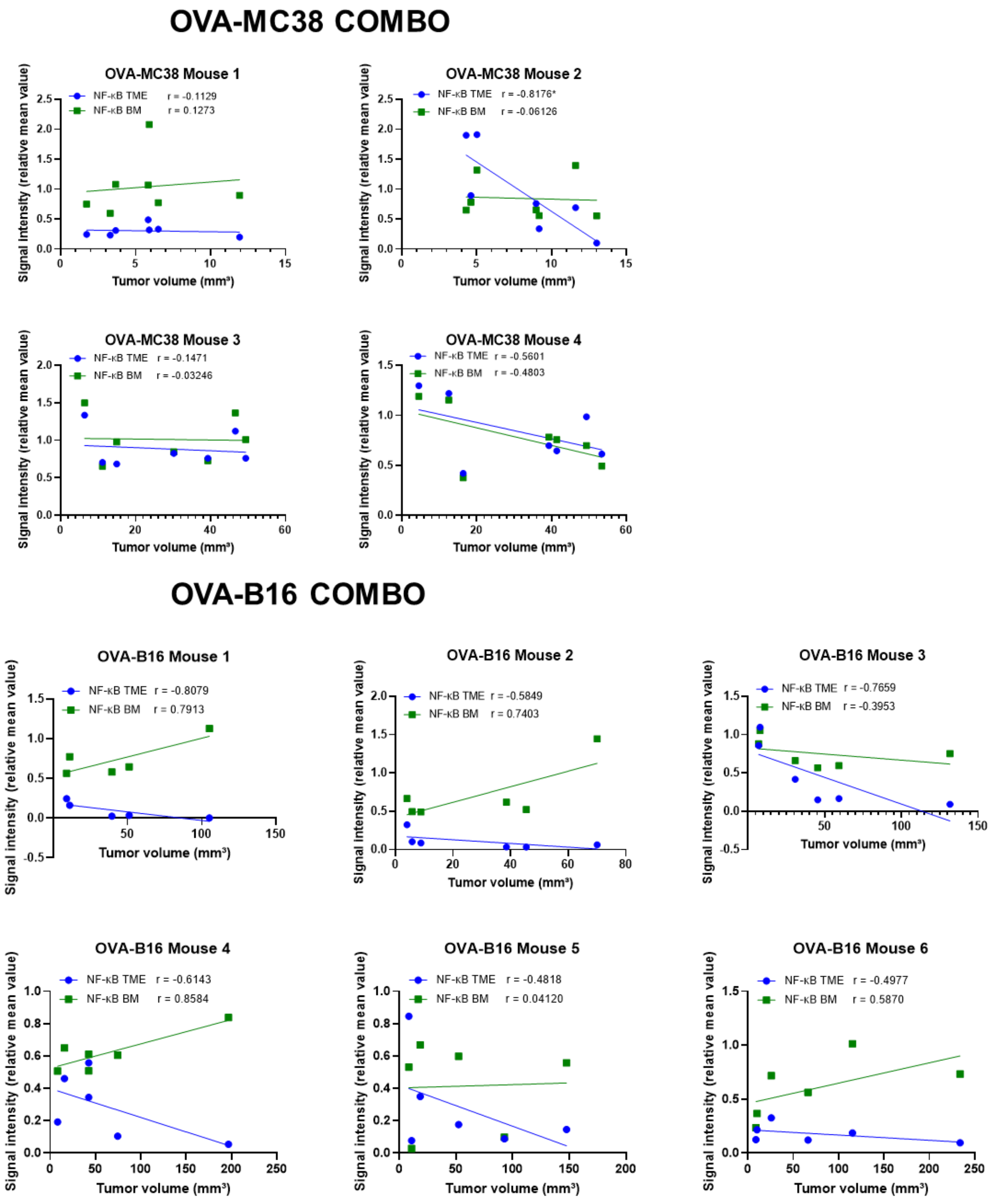

Supplemental Figure 9: Intra-individual correlation plots. Correlation of Tumor size and relative BLI-SI in the TME and BM over time (days 5–10) in COMBO-treated OVA-MC38 and OVA-B16 tumor-bearing mice. Pearson correlation coefficients (two-tailed).

Supplemental Table 1

Primary antibody	Secondary antibody
Goat-anti-Luciferase<br>(Novusbio NB100-1677)	EAZ-Alexa647<br>(Dianova, 705-606-147)
Rabbit-anti-CD3<br>(DCS, C1597C01)	EAK-Cy3<br>(Dianova, 711-166-152)
Rabbit-anti-NF-kB p50<br>(Invitrogen 51-3500)	
Rabbit-anti-MPO<br>(Dako A0389)	

The ARRIVE Essential 10: Compliance Questionnaire

Use this questionnaire to evaluate how well a manuscript complies with the ARRIVE Essential 10. It can be applied to any manuscript describing comparative experiments in living animals, by assessors such as journal staff, editors, or peer reviewers.

Item		Question(s)	Answers
1	Study Design	Are all experimental and control groups clearly identified?	☒ Yes, for at least one experiment<br>☐ No
		Is the experimental unit (e.g. an animal, litter or cage of animals) clearly identified?	☒ Yes, for at least one experiment<br>☐ No
2	Sample Size	Is the exact number of experimental units in each group at the start of the study provided (e.g. in the format '*n*=')?	☒ Yes, for at least one experiment<br>☐ No
		Is the method by which the sample size was chosen explained?	☒ Yes, for at least one experiment<br>☐ No
3	Inclusion & Exclusion Criteria	Are the criteria used for including and excluding animals, experimental units, or data points provided?	☒ Yes, for at least one experiment<br>☐ No
		Are any exclusions of animals, experimental units, or data points reported, or is there a statement indicating that there were no exclusions?	☒ Yes, for at least one analysis<br>☐ No
4	Randomisation	Is the method by which experimental units were allocated to control and treatment groups described?	☒ Yes, for at least one experiment<br>☐ No
5	Blinding	Is it clear whether researchers were aware of, or blinded to, the group allocation at any stage of the experiment or data analysis?	☒ Yes, for at least one experiment<br>☐ No
6	Outcome Measures	For all experimental outcomes presented, are details provided of exactly what parameter was measured?	☒ Yes, for at least one experiment<br>☐ No
7	Statistical Methods	Is the statistical approach used to analyse each outcome detailed?	☒ Yes, for at least one analysis<br>☐ No
		Is there a description of any methods used to assess whether data met statistical assumptions?	☐ Yes, for at least one analysis<br>☐ No<br>☒ Not applicable
8	Experimental Animals	Are all species of animal used specified?	☒ Yes, for at least one experiment<br>☐ No
		Is the sex of the animals specified?	☒ Yes, for at least one experiment<br>☐ No<br>☐ Not applicable to species
		Is at least one of age, weight or developmental stage of the animals specified?	☒ Yes, for at least one experiment<br>☐ No
9	Experimental Procedures	Are both the timing and frequency with which procedures took place specified?	☒ Yes, for at least one experiment<br>☐ No
		Are details of acclimatisation periods to experimental locations provided?	☒ Yes, for at least one experiment<br>☐ No
10	Results	Are descriptive statistics for each experimental group provided, with a measure of variability (e.g. mean and SD, or median and range)?	☒ Yes, for at least one experiment<br>☐ No<br>☐ Not applicable to the type of data collected
		Is the effect size and confidence interval provided?	☐ Yes, for at least one experiment<br>☐ No<br>☒ Not applicable to the type of analysis used
